# Decreased Gas6 and sAxl Plasma Levels Are Associated with Hair Loss in COVID-19 Survivors

**DOI:** 10.3390/ijms24076257

**Published:** 2023-03-26

**Authors:** Daria Apostolo, Davide D’Onghia, Stelvio Tonello, Rosalba Minisini, Alessio Baricich, Carla Gramaglia, Filippo Patrucco, Patrizia Zeppegno, Antonio Acquaviva, Piero Emilio Balbo, Luigi Mario Castello, Giuseppe Cappellano, Annalisa Chiocchetti, Chiara Gerevini, Mara Giordano, Fatiha Laaguid, Marcello Manfredi, Davide Raineri, Cristina Rigamonti, Roberta Rolla, Valentina Romano, Marco Confalonieri, Paola Savoia, Elisa Zavattaro, Mario Pirisi, Barbara Ruaro, Pier Paolo Sainaghi, Mattia Bellan

**Affiliations:** 1 Department of Translational Medicine, Università del Piemonte Orientale (UPO), 28100 Novara, Italy; 2 Center for Autoimmune and Allergic Disease (CAAD), Università del Piemonte Orientale (UPO), 28100 Novara, Italy; 3 A.O.U. Maggiore della Carità, 28100 Novara, Italy; 4 Unit of Internal Medicine, Azienda Ospedaliera “SS Antonio e Biagio e Cesare Arrigo”, 15121 Alessandria, Italy; 5 Pulmonology Department, University of Trieste, 34128 Trieste, Italy; 6 Department of Health Science, Università del Piemonte Orientale (UPO), 28100 Novara, Italy

**Keywords:** post-acute COVID-19, Gas6, TAM receptor, inflammation, hair loss

## Abstract

Post-acute conditions after coronavirus disease 2019 (COVID-19) are quite common, although the underlying pathogenetic mechanisms leading to these conditions are not yet completely understood. In this prospective observational study, we aimed to test the hypothesis that Growth Arrest-Specific 6 (Gas6) and its soluble receptors, Axl (sAxl) and MerTK (sMer), might be implicated. A total of 263 subjects underwent a structured clinical evaluation one year after their hospital discharge for COVID-19, and they consented to donate a blood sample to measure their circulating Gas6, sAxl, and sMer levels. A total of 98 (37.3%) post-COVID-19 subjects complained of at least one residual physical symptom one year after their hospital discharge. Univariate analysis revealed that sAxl was marginally associated with residual symptoms, but at the level of logistic regression analysis, only the diffusing capacity of the lungs for carbon monoxide (DLCO) (OR 0.98, CI 95%: 0.96–0.99; *p* = 0.007) and the female sex (OR 2.49, CI 95%: 1.45–4.28; *p* = 0.001) were independently associated with long-lasting symptoms. A total of 69 (26.2%) subjects had hair loss. At the level of univariate analysis, Gas6, sAxl, DLCO, and the female gender were associated with its development. In a logistic regression analysis model, Gas6 (OR 0.96, CI 95%: 0.92–0.99; *p* = 0.015) and sAxl (OR 0.98, CI 95%; 0.97–1.0; *p* = 0.014), along with the female sex (OR 6.58, CI 95%: 3.39–12.78; *p* = 0.0001), were independent predictors of hair loss. Decreased levels of Gas6 and sAxl were associated with a history of hair loss following COVID-19. This was resolved spontaneously in most patients, although 23.7% complained of persistent hair loss one year after hospital discharge.

## 1. Introduction

It is widely acknowledged that a relevant proportion of coronavirus disease 2019 (COVID-19) survivors continue to experience physical and neuropsychiatric symptoms including fatigue, dyspnea, chest tightness, cough, “brain fog”, headache, sleep difficulties, anxiety, and depression for several months after the initial recovery [1,2,3,4]. Less frequently, patients could also develop osteoarticular complaints and neurosensorial alterations [5]. These post-acute conditions, also known as “Long COVID”, include a constellation of signs and debilitating symptoms that remain or develop after Severe Acute Respiratory Syndrome Coronavirus 2 (SARS-CoV-2) infection has seemingly resolved, lasting for more than 2 months and remaining unexplained by an alternative diagnosis [6,7,8]. At present, the underlying processes involved in long COVID pathogenesis are not fully understood [9]. However, it has been suggested that the persistence of a viral reservoir and the establishment of a pro-coagulant condition driven by SARS-CoV-2 infection, together with a chronic pro-inflammatory status promoted by cytokines, may lead to these long-lasting symptoms [10,11,12,13].

Growth arrest-specific 6 (Gas6) and its receptors, Axl and MerTK, are known to play a major role in promoting and maintaining inflammation [14]. Gas6 is a 75 kDa soluble glycoprotein that belongs to the family of vitamin-K-dependent proteins and interacts with TAM, a specific tyrosine kinase (TKr) receptor family consisting of three different receptors: Tyro-3, Axl, and MerTK [15,16]. Gas6 binds Axl with a higher affinity than when compared to Tyro-3 and MerTK [17,18]. After the cleavage by proteases, the extracytoplasmic structure of the transmembrane receptor is released into the plasma in a soluble form (sAxl and sMer) that is still able to bind Gas6, and it is probably exerting a modulatory function [19]. Gas6/TAM signaling is a highly pleiotropic system involved in cell survival, growth, aggregation and migration, angiogenesis, and in the control of inflammatory responses [20,21,22]. Moreover, the system is engaged in chronic immunological diseases, with its overactivation linked to several neoplastic conditions [23,24,25,26,27,28,29,30,31,32,33,34]. More recently, the TAM system has been proposed as involved in COVID-19. Indeed, different authors have suggested the importance of the TAM receptor family in SARS-CoV-2 infection, COVID-19 severity, and the development of clinical complications. In particular, it has been reported that higher baseline plasma Gas6 and sAxl concentrations are associated with worsening clinical conditions and that they correlate directly with disease severity [35,36]. In addition, it has been highlighted that TAM signaling could be involved in coagulopathies correlated to aberrant inflammation and fibrosis development in COVID-19 patients [37,38].

While the involvement of Gas6/TAM signaling has already been investigated in the acute phase of the disease, its role in the development of long COVID syndrome remains unexplored. Thus, the aim of the present study was to investigate a possible association between circulating Gas6, sAxl, and sMer levels with long-term sequelae in COVID-19 survivors.

## 2. Results

### 2.1. Study Population

A total of 263 patients previously hospitalized for COVID-19 (162 males, 61.6%), with a median age of 60 (IQR: 51–69) years old, were recruited for this study. During the acute phase of the disease, the median duration of the hospital stay was 11 days [7,8,9,10,11,12,13,14,15,16], the median cumulative illness rating scale (CIRS) in the study population was 2 [1,2,3,4], and the class of severity was distributed as shown in Table 1.

### 2.2. Residual Symptoms

After a median period of 368 (IQR: 364–392) days, 98 (37.3%) subjects complained about residual symptoms, as reported in Supplementary Figure S1.

First, we evaluated whether Gas6/sTAM values, along with clinical variables of potential interest according to our previous publications on the same cohort [3,5,13], were associated with the persistence of physical symptoms 1 year after hospital discharge. Data are shown in Supplementary Table S1.

**Figure 1:**
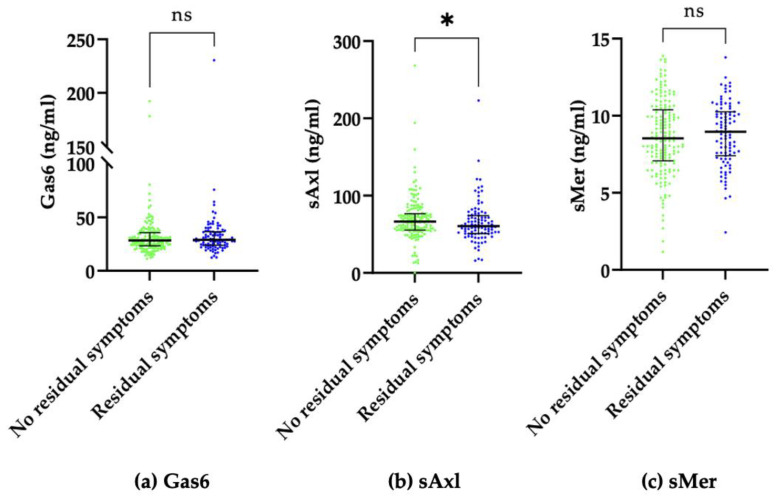
Comparison between median plasma Gas6 concentration (**a**), sAxl concentration (**b**), and sMer concentration (**c**) of patients with and without residual symptoms one year after hospital discharge. Results are shown as medians (IQR). * *p* = 0.036, ns: not significant.

As reported in Supplementary Table S1, at univariate analysis, only sAxl was significantly lower in patients complaining of persistent symptoms (Figure 1). We then built a multivariate model with variables that were significantly associated (*p* < 0.05) with univariate analysis, which included DLCO, the female sex, and sAxl plasma concentrations. As shown in Table 2, sAxl lost its statistical significance. In addition, we discovered that both sAxl (*p* < 0.008) and Gas6 (*p* < 0.005) but not sMer (*p* < 0.09) levels one year after hospital discharge were directly associated with the class of severity reported during the acute phase of the disease.

### 2.3. Hair Loss

Moreover, 26.2% (N. = 69) of the study population reported hair loss following their contraction of SARS-CoV-2 infection.

**Figure 2:**
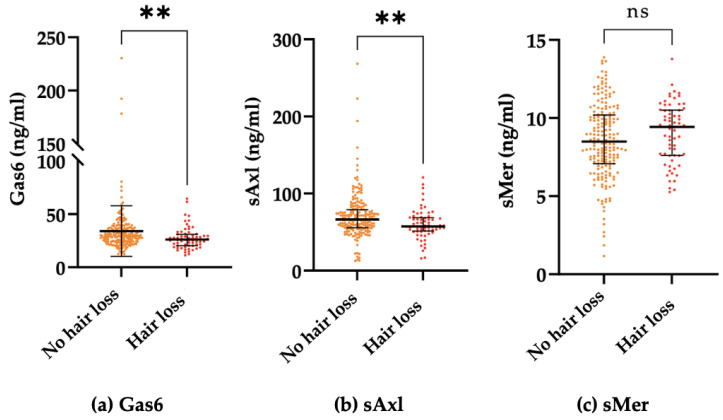
Comparison between median plasma Gas6 concentration (**a**), sAxl concentration (**b**), and sMer concentration (**c**) of patients reporting hair loss and patients without hair loss. Results are shown as medians (IQR). ** *p* = 0.001, ns not significant.

We also evaluated the association of the Gas6/sTAM system with the development of hair loss (Table 3). Interestingly, both Gas6 and sAxl significantly decreased in patients describing hair loss who first complained after experiencing SARS-CoV-2 infection (Figure 2).

At univariate analysis, Gas6, sAxl, DLCO, and the female sex were associated with the development of hair loss (*p* < 0.05). We then built a logistic regression analysis including the variables of Gas6, sAxl, and the female sex, which maintained their significant association with hair loss; however, DLCO did not fit into the model (Table 4).

Ultimately, to better elucidate the nature and persistence of hair loss, the subjects were retrospectively invited to answer a short questionnaire, and 59/69 (85.5%) subjects agreed to participate. The results are extensively reported in Supplementary Table S2. Interestingly, 64.4% of the subjects complaining of hair loss reported that they first noticed it months after their hospital discharge. Moreover, 74.6% of subjects reported acute/massive hair loss that was largely resolved by the time of the interview. Furthermore, 71.2% of subjects reported that their hair loss was completely resolved within 1 year from their hospital discharge, and this increased to 76.3% by the time of the phone interview. More commonly, hair loss was an isolated incident. Although, around 1/3 of patients complained of associated symptoms, such as an itching or burning sensation. Finally, hair loss was generally diffuse (45.8%). However, it was reported in some cases to be more localized to the forehead (23.7%), the top of the head (20.3%), or was patchy (10.2%). In addition, 5 (8.5%) participants declared to have lost not only hair on their scalp but also their eyelashes, eyebrows, or beard, and 22 (37.3%) participants reported a slowdown in their hair growth rate.

## 3. Discussion

While COVID-19 is still a global public health threat, the persistence of symptoms and long-term sequelae in people who recovered from SARS-CoV-2 acute infection is emerging as a relevant health issue worldwide. In this study, we aimed to evaluate the potential implication of the Gas6/TAM system in the pathogenesis of long COVID. Based on our data, this system does not seem to be altered among patients complaining of post-acute COVID symptoms—with a single major exception: hair loss after SARS-CoV-2 infection.

Hair loss is common among COVID-19 survivors. In the present cohort, more than one-quarter of subjects complained of hair loss following COVID-19, which is a figure in line with what was reported in a recent meta-analysis reporting that the prevalence of hair loss in COVID-19 survivors is 25% (CI95%: 17–34%) [39].

The mechanisms underlying this specific complication are unknown. Several risk factors have been suggested as major players, such as the stress induced by a viral infection, the potential involvement of the treatment(s) used during the acute phase, and the psychosocial stress induced by the global pandemic itself [40].

To elaborate, chronic stress seems to impact hair growth by keeping hair follicle stem cells (HFSCs) in a quiescent state due to microenvironment changes with the consequent hair loss [41]. Although the Gas6 gene has been revealed as a candidate gene working as a regulator of hair growth, given the fact that it is stress sensitive, there is currently a paucity of data evaluating the involvement of this system in the development of hair loss. In a recent paper, Choi et al. demonstrated that corticosterone, which is the rodent equivalent of cortisol in humans, controls HFCS’s quiescence and hair growth in mice via the regulation of Gas6 expression. It is worth noting that the Gas6 protein in normal conditions is able to bind the Axl receptor on HFSCs activating the genes involved in the cell cycle [42]. Under chronic stress conditions, increased corticosterone levels lead to the suppression of the Gas6 expression acting on the dermal papillae. In mice, the forced expression of Gas6 in the dermis is able to overcome this inhibition and promotes hair growth [43]. Therefore, according to these studies, higher levels of Gas6 may protect against hair loss. In line with this hypothesis is that in our study population, the patients complaining of hair loss showed decreased levels of Gas6 and sAxl compared to all others.

Although these findings are interesting, our study has some limitations. First, the population size is relatively small. Second, we did not measure the baseline levels of Gas6 and sTAM to understand whether these biomarkers may also have a pathogenetic role in the development of hair loss. Lastly, the definition of hair loss was self-reported without a formal hair loss diagnosis. Indeed, to overcome the lack of information about this specific complaint, we administered a retrospective questionnaire. According to this, the majority of patients described diffuse hair loss, and only a minority described patchy hair loss. In more than 75% of subjects, the symptom was completely resolved over time; although, around a quarter reported persistent hair loss. Regarding patients who recovered, a possible explanation could be telogen effluvium (TE), which is characterized by diffuse hair loss and increased hair shedding after several weeks post-infection. In a recent systemic review and meta-analysis, Nguyen and Tosti described the hair-related manifestations of COVID-19, including telogen effluvium, which is presented as a new-onset sequela of COVID-19 [44]. Moreover, as suggested by Christensen et al., alopecia areata may also be a dermatologic manifestation that arises 1 to 2 months following a contraction of SARS-CoV-2 infection [45].

We reported that the female sex is strongly associated with hair loss, as increased hair shedding is more noticeable in women compared to men. However, our findings strengthen the rationale basis for further studies evaluating the potential involvement of this pleiotropic system in hair loss beyond the setting of the SARS-CoV-2 infection.

We also evaluated the potential association of Gas6 and its soluble receptors with long COVID. This hypothesis was based on the observation that long COVID may be associated with persistent organ damage and lung fibrosis [5]; moreover, according to recent evidence, this condition may be associated with the presence of persistent low-grade inflammation, as disclosed by increased levels of pro-inflammatory cytokines [46]. Gas6 is a central molecule in the regulation of the interplay between inflammation and fibrosis [14]; therefore, it is a candidate biomarker of long COVID with a strong rationale. The prevalence of persistent complaints was high in our population (39.3%), and this was consistent with the data that we already published on a larger cohort at the same time point, to which this subset of subjects belongs [5]. However, only sAxl levels were marginally decreased in long COVID patients in our population, and this association was lost when considered in the context of a multivariate model. This seems to rule out a potential role of this system; however, as mentioned above, the baseline levels of Gas6/sTAM should be assessed to definitively rule out a potential role of this system. Indeed, in previous papers, higher plasma Gas6 concentrations upon hospital admission during the acute phase of COVID-19 predicted a more severe evolution [35,36]. Whether this is also associated with an increased risk of long-term sequelae development should be assessed in an ad hoc study. It should be noted, however, that in our cohort the severity of acute disease was not predictive of long COVID development. Lastly, it is also worth noting that Gas6 and sAxl levels one year after hospital discharge remain associated with the class of severity reported in the acute phase of COVID-19, as this was already shown in baseline Gas6 and sAxl levels by other authors [36].

In conclusion, our findings confirm that hair loss is common among COVID-19 survivors and primarily suggest that this symptom may be associated with a derangement of the Gas6/TAM system. Indeed, while the levels of Gas6 and its soluble receptors one year after the acute disease bear no significant association with persistent organic symptoms, lower Gas6 and sAxl are associated with patients with a history of hair loss following COVID-19. A deeper understanding of the possible factors that contribute to hair loss in COVID-19 survivors could provide novel insights about this common condition in the general population, possibly identifying a novel and relevant pathway.

## 4. Methods and Materials

### 4.1. Patients

In this prospective observational cohort study, 263 COVID-19 survivors were enrolled in the “Maggiore della Carità” Hospital in Novara, Italy. The data presented in this paper belong to a larger cohort study, the results of which have been partially reported in previous publications [3,5]. A convenience sample of subjects who survived hospitalization for SARS-CoV-2 infection between March 2020 and June 2021 during the first and third Italian wave was invited to attend a follow-up visit 12 months after their hospital discharge. Follow-up visits have been carried out from March 2021 to June 2022 in a multidisciplinary clinic. All participants signed an informed consent form and were included if they were over 18 years old and had a diagnosis of COVID-19 confirmed during their hospital stay using a reverse transcription polymerase chain reaction (RT-PCR) with a nasopharyngeal swab.

The study protocol was approved by the local Ethical Committee (CE 117/20) and was conducted in strict accordance with the Declaration of Helsinki.

### 4.2. Clinical Evaluation

Data about patients’ demographic characteristics, ongoing drugs, symptoms at COVID-19 diagnosis and follow-up evaluation, complications during their hospital stay, and their type and number of comorbidities were collected by clinicians involved in the treatment of patients. Among the signs and symptoms investigated, we recorded whether the patients experienced hair loss in the follow-up period. These subjects were invited to answer a short retrospective questionnaire about the nature and persistence of hair loss. The severity of the acute phase of the disease was classified using an eight-category scale, as previously described [47]. Moreover, all patients underwent standard pulmonary function testing (PFT) with a Quark PFT with X9 pneumotach (COSMED) for assessing the forced expiratory volume in 1 s (FEV1), vital capacity, forced vital capacity (FVC), and diffusion capacity of the lung for carbon monoxide (DLCO), and the total lung capacity was determined by the single-breath CO technique.

### 4.3. Blood Sample Collection

Blood was collected by venous punctures using EDTA as an anticoagulant. Plasma was immediately collected by centrifugation at 3500 rpm for 15 min and stored at −80 °C until the time of analysis. This research has been conducted using UPO Biobank Resources.

### 4.4. Circulating Gas6 Levels Determination

The plasma levels of Gas6 were determined by the ELISA technique by using a commercial kit (R&D Systems DuoSet Elisa DY6488, McKinley, MN, USA) and following the manufacturer’s instructions. Prior to Gas6 quantification, plasma samples were diluted 1:50. Absorbance was recorded using a Victor X4 microplate reader (Perkin Elmer, Waltham, MA, USA). The optical density at 450 nm was fitted versus a calibration curve that was prepared with a standard (0 ng/mL–1 ng/mL range), as suggested by the manufacturer.

### 4.5. Soluble Axl (sAxl) Levels Determination

Plasma levels of sAxl were determined by the ELISA technique by using a commercial kit (R&D Systems DuoSet Elisa DY6488, McKinley, MN, USA) and following the manufacturer’s instructions. Prior to sAxl quantification, plasma samples were diluted 1:50 in PBS. Absorbance was recorded using a Victor X4 microplate reader (Perkin Elmer, Waltham, MA, USA). The optical density at 450 nm was fitted versus a calibration curve prepared with a standard (0 ng/mL–4 ng/mL range), as suggested by the manufacturer.

### 4.6. Soluble Mer (sMer) Levels Determination

The plasma levels of sMer were determined by the ELISA technique by using a commercial kit (R&D Systems DuoSet Elisa DY6488, McKinley, MN, USA) and following the manufacturer’s instructions. Absorbance was recorded using a Victor X4 microplate reader (Perkin Elmer, Waltham, MA, USA). The optical density at 450 nm was fitted versus a calibration curve prepared with a standard (0 ng/mL–10 ng/mL range), as suggested by the manufacturer.

### 4.7. Statistical Analysis

For continuous variables, the measures of centrality and dispersion were medians and interquartile ranges (IQR), and comparisons between groups regarding these variables were performed using the Mann–Whitney U test. The Pearson χ^2^ or Fisher’s exact test was used, as appropriate, to analyze the association between categorical variables that are shown as frequencies (%).

Multivariable stepwise regression models were built to identify the variables independently associated with the persistence of symptoms one year after hospital discharge. The threshold for statistical significance was 0.05 (two-tailed). Statistical analyses were performed with Stata statistical software version 17.0 (StataCorp, 4905 Lakeway Drive College Station, TX, USA) and MedCalc^®^ Statistical Software version 20.113 (MedCalc Software Ltd., Ostend, Belgium), while graphs were created using GraphPad Prism version 9.4.0 (GraphPad Software, La Jolla, CA, USA).

## Figures and Tables

**Table 1:** Distribution for the class of severity in the study population at their time of hospitalization.

Class of Severity	Frequency (%)
3	40 (15.2)
4	5 (1.9)
5	86 (32.7)
6	115 (43.7)
7	17 (6.5)

**Table 2:** Variables associated with the persistence of symptoms one year after hospital discharge. Coefficient, *p*-value; the odds ratio (OR) with a 95% confidence interval (CI) is reported. The bold text highlights statistically significant results. For abbreviation: DLCO, diffusing capacity of carbon monoxide in lungs.

Predictors	Coefficient	*p*-Value	OR (95%CI)
Sex (female)	0.91	**0.001**	2.49 (1.45–4.28)
DLCO, %	−0.02	**0.007**	0.98 (0.96–0.99)
sAxl (ng/mL)	−0.006	0.24	0.99 (0.98–1.0)

**Table 3:** Univariate analysis of clinical variables and Gas6/sTAM among patients with and without hair loss. Evaluation of the possible association between hair loss and clinical variables: CIRS, DLCO, age, Gas6, sAxl, sMer, sex, and class of severity. Continuous variables are presented as medians and interquartile range (IQR), while categorical variables are an absolute number (%). Bold text highlights statistically significant results. For abbreviation: CIRS, cumulative illness rating scale; DLCO, diffusing capacity of carbon monoxide in lungs.

Variables		No Hair Loss (n = 194)	Hair Loss (n = 69)	*p*-Value
CIRS		2.0 (1–4)	2.0 (1–3)	0.10
DLCO, %		82.0 (70–93)	76.0 (68–85)	**0.017**
Age, years		59 (51–68)	61 (50–70)	0.38
Gas6 (ng/mL)		29.39 (24.2–36.8)	26.01 (20.3–30.9)	**0.0012**
sAxl (ng/mL)		66.5 (55.6–79.1)	57.3 (51.6–68.1)	**0.0011**
sMer (ng/mL)		8,49 (7.09–10.18)	9.43 (7.61–10.49)	0.12
Sex (male/female)		141 (72.7)/53 (27.3)	21 (30.4)/48 (69.6)	**0.0001**
Class of severity	3	29 (15)	11 (15.9)	0.78
	4	4 (2)	1 (1.5)	
	5	62 (32)	24 (34.8)	
	6	91 (46.9)	24 (34.8)	
	7	8 (4.1)	9 (13)	

**Table 4:** Variables associated with the development of hair loss. Coefficient, *p*-value, and odds ratio (OR) with a 95% confidence interval are reported in the table. The bold text highlights the statistically significant results. For abbreviation: DLCO, diffusing capacity of carbon monoxide in lungs.

Predictors	Coefficient	*p*-Value	OR (95%CI)
Sex (female)	1.88	**0.0001**	6.58 (3.39–12.78)
DLCO, %	−0.01	0.35	0.99 (0.97–1.01)
Gas6 (ng/mL)	−0.04	**0.015**	0.96 (0.92–0.99)
sAxl (ng/mL)	−0.02	**0.014**	0.98 (0.97–1.0)

## Data Availability

M.B. has full access to all the data of the study and takes responsibility for the integrity of the data and the accuracy of the data analysis.

## Supplementary Materials

The supporting information can be downloaded at https://www.mdpi.com/article/10.3390/ijms24076257/s1.

## Appendix A

No-more COVID group: Antonio Acquaviva, Alice Albè, Daria Apostolo, Piero Emilio Balbo, Giulia Baldon, Alessio Baricich, Michela Barini, Marco Battaglia, Simone Bor, Vincenzo Cantaluppi, Giuseppe Cappellano, Alessandro Carriero, Sara Casella, Luigi Mario Castello, Annalisa Chiocchetti, Elisa Clivati, Donato Colangelo, Marco Confalonieri, Martina Crevola, Daria Cuneo, Davide D’Onghia, Nicolò Errica, Eleonora Gambaro, Chiara Gerevini, Mara Giordano, Carla Gramaglia, Luisa Isabella, Fatiha Laaguid, Alberto Loro, Marcello Manfredi, Debora Marangon, Rosalba Minisini, Emanuele Mones, Elena Paracchini, Filippo Patrucco, Giuseppe Patti, David James Pinato, Mario Pirisi, Chiara Puricelli, Davide Raineri, Giacomo Ratano, Cristina Rigamonti, Roberta Rolla, Valentina Romano, Barbara Ruaro, Paola Savoia, Stelvio Tonello, Stefano Tricca, Elisa Zavattaro, Patrizia Zeppegno, Pier Paolo Sainaghi and Mattia Bellan.
