# Supplementary material for: Decreased Gas6 and sAxl Plasma Levels Are Associated with Hair Loss in COVID-19 Survivors
Source: Int J Mol Sci. 2023 Mar 26;24(7):6257. doi: 10.3390/ijms24076257 (PMC10094682; doi:10.3390/ijms24076257)
Supplement: Supplementary file 1 [file ijms-24-06257-s001.zip › ijms-2292680-supplementary.pdf]

SUPPLEMENTARY

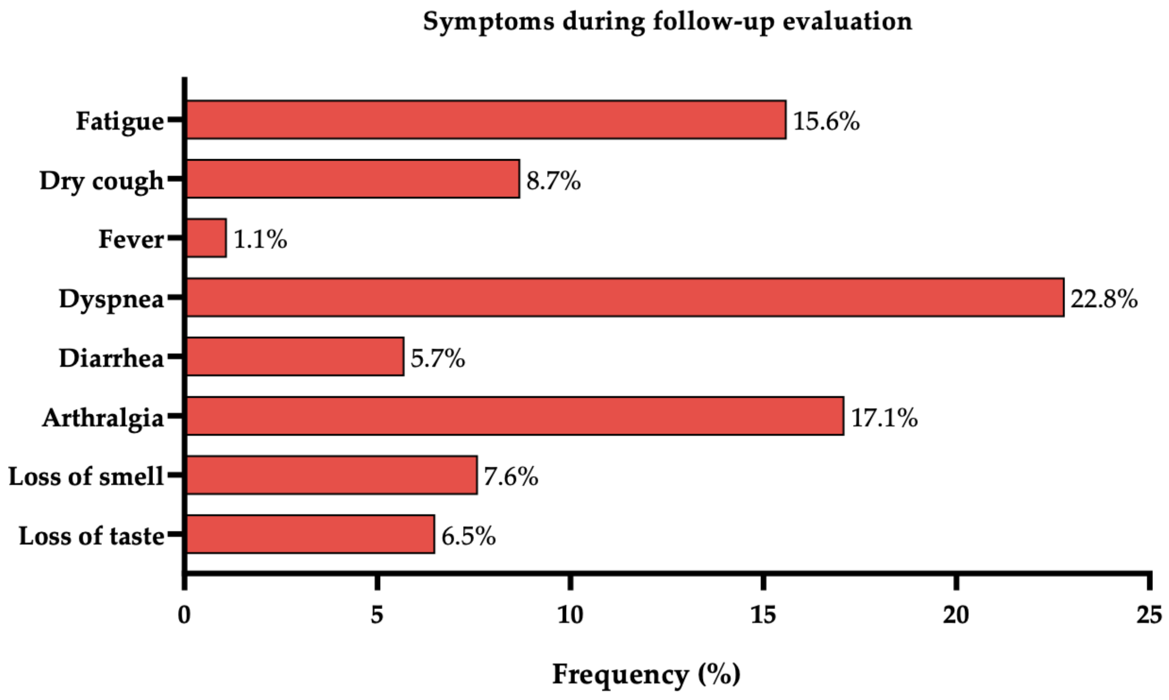

Supplementary Figure S1: **Symptoms during the follow-up evaluation.** The graph shows the frequency of the most common symptoms during the follow-up visits expressed as percentage.

Supplementary Table S1: **Univariate analysis of clinical variables and Gas6/sTAM among patients with and without residual symptoms.** Evaluation of possible association between residuals symptoms at one year follow-up visits and clinical variables: CIRS, DLCO, age, Gas6, sAxl, sMer, gender and class of severity. Continuous variables are presented as medians and interquartile range [IQR], while categorical variables as an absolute number (%). Bold text highlights the statistically significant results. For abbreviation: CIRS, cumulative illness rating scale; DLCO, diffusing capacity of carbon monoxide in lungs.

**VARIABLES**	**NO RESIDUAL SYMPTOMS (n=165)**	**RESIDUAL SYMPTOMS (n=98)**	***p*-value**
	Median [IQR]	Median [IQR]	
CIRS	2.0 [1-4]	2.0 [2-4]	0.43
DLCO, %	83.0 [72-96]	76.0 [66-84]	**0.0002**
Age, years	60 [51-67]	61 [50-70]	0.47
Gas6 (ng/ml)	28.36 [23.4-35.63]	28.90 [24-36,47]	0.37
sAxl (ng/ml)	66.44 [55.59-76.65]	60.60 [50.9-74.1]	**0.036**
sMer (ng/ml)	8.53 [7.07-10.38]	8.96 [7.4-10.25]	0.49

Sex (male/female)		116 (70.3%)/49 (29.7%)	46 (46.9%)/52 (53.1%)	**0.0002**
Class of severity	3	24 (14.5%)	16 (16.3%)	0.64
	4	3 (1.8%)	2 (2%)	
	5	59 (35.8%)	27 (27.6%)	
	6	71 (43%)	44 (44.9%)	
	7	8 (4.9%)	9 (9.2%)	
Symptoms at the follow-up evaluation		165 (62.7%)	98 (37.2%)	**0.0001**

Supplementary Table S2: **Results of the questionnaire assessing the features of hair loss.**

**When did you start notice hair loss?**	
1. Early after hospital discharge<br>2. Months after hospital discharge	21/59 (35.6%)<br>38/59 (64.4%)
**Type of hair loss**	
1. Acute/massive<br>2. Chronic	44/59 (74.6%)<br>15/59 (25.4%)
**Characteristics of hair loss**	
1. Increased number of falling hair<br>2. Thinning hair without increased of falling hair	36/59 (78.0%)<br>13/59 (22.0%)
**How did you observed hair loss?**	
1. While washing your hair<br>2. While drying your hair<br>3. While combing your hair<br>4. Presence of hair on the bathroom floor<br>5. Presence of hair on the pillow in the morning	23/59 (39.0%)<br>1/59 (1.7%)<br>23/59 (39.0%)<br>1/59 (1.7%)<br>11/59 (18.6%)
**How long did you notice hair loss?**	
1. Less than 1 year<br>2. More than 1 year	42/59 (71.2%)<br>17/59 (28.8%)
**Concomitant pathologies**	
1. No one<br>2. Thyroiditis<br>3. Vitiligo<br>4. Polycystic ovary	50/59 (84.7%)<br>7/59 (11.9%)<br>0/59 (0%)<br>2/59 (3.4%)
**Previous diagnosis or treatments for alopecia**	
1. Yes<br>2. No	2/59 (3.4%)<br>57/59 (96.6%)
**Family history for alopecia**	
1. Yes<br>2. No	5/59 (8.5%)<br>54/59 (91.5%)
**Concomitant or previous therapies**	
1. No one<br>2. Chemotherapy or immunotherapy	44/59 (74.5%)<br>1/59 (1.7%)

3. Anticoagulants<br>4. Antiepileptic<br>5. Beta-blockers<br>6. Tricyclic antidepressant<br>7. Anticoagulants and beta-blocker together	3/59 (5.1%)<br>0/59 (0%)<br>7/59 (11.9%)<br>1/59 (1.7%)<br>3/59 (5.1%)
**Nutritional deficiencies**	
1. No one<br>2. Supplementation of iron<br>3. Supplementation of calcium<br>4. Supplementation of Vitamin D<br>5. Supplementation of calcium and vitamin D	26/59 (44.0%)<br>2/59 (3.4%)<br>1/59 (1.7%)<br>27/59 (45.8%)<br>3/59 (5.1%)
**Area of interest**	
1. Forehead<br>2. On top of the head<br>3. Spread all over the head<br>4. Patches on the head	14/59 (23.7%)<br>12/59 (20.3%)<br>27/59 (45.8%)<br>6/59 (10.2%)
**Characteristics of falling hair**	
1. Normal<br>2. Thinned<br>3. Broken	37/59 (62.7%)<br>20/59 (33.9%)<br>2/59 (3.4%)
**Slowdown in hair growth rate**	
1. Yes<br>2. No	22/59 (37.3%)<br>37/59 (62.7%)
**Other area of interest**	
1. No one<br>2. Eyelashes<br>3. Eyebrows<br>4. Beard	54/59 (91.5%)<br>3/59 (5.1%)<br>1/59 (1.7%)<br>1/59 (1.7%)
**Presence of lesions on the scalp**	
1. No one<br>2. Pustules<br>3. Scales<br>4. Atrophic areas	51/59 (86.4%)<br>4/59 (6.8%)<br>3/59 (5.1%)<br>1/59 (1.7%)
**Visual perception of thinning**	
1. Only by participant him/herself<br>2. By Participant and other people	21/59 (35.6%)<br>38/59 (64.4%)
**Impact on quality of life**	
1. Yes<br>2. No	18/59 (30.5%)<br>41/59 (69.5%)
**Necessity to hide the hair loss**	
1. Yes<br>2. No	8/59 (13.6%)<br>51/59 (86.4%)
**Associated symptoms**	
1. No one<br>2. Itching<br>3. Pain<br>4. Burning<br>5. Hyperesthesia	40/59 (67.8%)<br>12/59 (20.3%)<br>1/59 (1.7%)<br>5/59 (8.5%)<br>1/59 (1.7%)
**Treatment**	
1. No one<br>2. Topic<br>3. Systemic	47/59 (79.6%)<br>5/59 (8.5%)<br>7/59 (11.9%)

Duration of treatment	
1. 1 month	2/12 (16.7%)
2. 2 months	2/12 (16.7%)
3. 3 months	2/12 (16.7%)
4. 4 months	2/12 (16.7%)
5. 6 months	4/12 (33.2%)
**Outcome**	
1. Resolved	45/59 (76.3%)
2. Not resolved	14/59 (23.7%)
